# Analysis of Target Vessel Instability in Fenestrated Endovascular Repair (f-EVAR) in Thoraco-Abdominal Aortic Pathologies

**DOI:** 10.3390/jcm13102898

**Published:** 2024-05-14

**Authors:** Daniel Becker, Laura Sikman, Ahmed Ali, Selim Mosbahi, Carlota F. Prendes, Jan Stana, Nikolaos Tsilimparis

**Affiliations:** 1 Department of Vascular Surgery, University Hospital, LMU Munich, 81377 Munich, Germany; dbeckermed@gmx.ch (D.B.); laurasikman@yahoo.de (L.S.); ahazhar92@live.com (A.A.); carlota.f.prendes@gmail.com (C.F.P.); jan.stana@med.uni-munechen.de (J.S.); 2 Department of Vascular Surgery, Cardiovascular and Vascular Surgery Center, University Hospital, Mansoura University, Mansoura 35516, Egypt; 3 Department of Cardiac Sugery, University Hospital, Inselspital Bern, 3010 Bern, Switzerland; selim.mosbahi@insel.ch

**Keywords:** fenestrated stent-grafts, complex abdominal aortic pathologies, thoraco-abdominal pathologies, target vessel instability

## Abstract

**Objective:** The aim of this study was to evaluate the influence of target vessel anatomy and post-stenting geometry on the outcome of fenestrated endovascular aortic repair (f-EVAR). **Methods:** A retrospective review of data from a single center was conducted, including all consecutive fenestrated endovascular aortic repairs (f-EVARs) performed between September 2018 and December 2023 for thoraco-abdominal aortic aneurysms (TAAAs) and complex abdominal aortic aneurysms (cAAAs). The analysis focused on the correlation of target vessel instability to target vessel anatomy and geometry after stenting. The primary endpoint was the cumulative incidence of target vessel instability. Secondary endpoints were the 30-day and follow-up re-interventions. **Results:** A total of 136 patients underwent f-EVAR with 481 stented target vessels. A total of ten target vessel instabilities occurred including three in visceral and seven instabilities in renal vessels. The cumulative incidence of target vessel instability with death as the competing risk was 1.4%, 1.8% and 3.4% at 1, 2 and 3 years, respectively. In renal target vessels (260/481), a diameter ≤ 4 mm (OR 1.21, 95% CI 1.035–1.274, *p* = 0.009) and an aortic protrusion ≥ 5.75 mm (OR 8.21, 95% CI 3.150–12-23, *p* = 0.027) was associated with an increased target vessel instability. In visceral target vessels (221/481), instability was significantly associated with a preoperative tortuosity index ≥ 1.25 (HR 15.19, CI 95% 2.50–17.47, *p* = 0.045) and an oversizing ratio of ≥1.25 (HR 7.739, CI % 4.756–12.878, *p* = 0.049). **Conclusions:** f-EVAR showed favorable mid-term results concerning target vessel instability in the current cohort. A diameter of ≤4 mm and an aortic protrusion of ≥5.75 mm in the renal target vessels as well as a preoperative tortuosity index and an oversizing of the bridging stent of ≥1.25 in the visceral target vessels should be avoided.

## 1. Introduction

Endovascular aortic repair with fenestrated endografts (f-EVAR) is a widely accepted treatment option for complex aortic pathologies. It has shown excellent outcomes concerning safety, technical success and patency rates of target vessels [1,2,3,4,5]. A crucial aspect of treatment is the connection of the main endograft to target vessels with bridging stent-grafts.

Since its introduction, f-EVAR procedures have been standardized over the years. There are different covered stent-grafts used as bridging stents in the field of f-EVAR. Despite numerous studies examining the use of bridging stent-grafts, to date no dedicated bridging stent-graft is available [6,7,8,9,10]. Promising results were derived from the group of Haulon et al. and our group reporting promising mid-term results with freedom from target vessel instability of 98–99% for BeGraft stent-grafts in f-EVAR [11,12].

The effect of target vessel anatomy before and after the placement of bridging stent-grafts on the outcome of f-EVAR is more and more under investigation. Concerning preoperative target vessel characteristics, it is well known that especially small renal arteries are at risk of instability in both f-EVAR and b-EVAR [13]. The reasons have not been clearly explained yet. An explanation might be that the available bridging stents have a minimal size of 5 mm, meaning an oversizing in 3–4 mm sized target vessels. Squizzato et al., for example, found a bridging length (gap) of ≥5 mm is associated with an increased target vessel instability [14]. This was also reported by the groups Chait and Oderich et al. [15,16].

In the field of fenestrated endovascular aneurysm repair (f-EVAR), there is a prevailing shift towards the use of balloon-expandable covered stents as bridging stents. This adaptation addresses the concern of how respiratory movements might affect target vessel deformation. In an illustrative study, Tran et al. evaluated the impact of respiratory dynamics on the performance of the Zenith f-EVAR system in combination with BeGraft peripheral covered bridging stents. Their research highlighted a decrease in flexibility, especially noticeable in renal target vessels. Nonetheless, this configuration still managed to closely mimic the native arterial geometry and accommodate the cyclical vessel deformation attributed to respiratory movements [17]. 

Despite these findings, there is still a lack of knowledge of the impact of target vessel anatomy on the outcome of f-EVAR.

The aim of this study was to evaluate the influence of target vessel anatomy and geometry after stenting on short- and midterm the outcome of f-EVAR.

## 2. Methods

All consecutive patients with TAAAs and cAAAs undergoing either urgent or elective f-EVAR between September 2018 and December 2023 were included. 

Preoperative demographics, clinical characteristics, cardiovascular risk factors, anatomical data and operative and postoperative variables were recorded prospectively in a dedicated database, and retrospectively analyzed. The anatomical extent of aortic aneurysm was classified according to the current reporting standards based on the preoperative CTA [16]. 

All procedures were performed under general anesthesia in a hybrid suite. Elective patients routinely underwent repair with custom-made Cook stent-grafts (Cook medical, Bloomington, NJ, USA). In patients undergoing urgent repair (symptomatic, ruptured, large aneurysm, etc.), a physician modified stent-graft using the Cook platform (Thoracic Alpha or TX2) or a modification of a CMD graft was performed. Procedural time, radiation dose, volume of contrast and radiation time were recorded. Procedural details included the presence of a lumbar drain, number of target vessels, type of surgical access and perioperative complications. Follow-up data were collected from the aortic outpatient clinical reports. All patients received a CT scan prior to discharge or during the first 30 days after the index procedure. Patients underwent clinical and radiological follow up examination in our aortic outpatient clinic, 6 months, 12 months and yearly thereafter. In patients with a GFR < 45 mL/min/1.73 m^2^, follow up was switched to contrast-enhanced ultrasound. Postoperative antithrombotic treatment consisted of double antiplatelet therapy for 6 months and, after an uncomplicated course, changed to lifelong monotherapy. In cases of additional necessary anticoagulation, only mono-antiplatelet therapy was administered. During follow up, data of major adverse events, target vessel instability, re-interventions and mortality were collected.

### 2.1. Target Vessel Anatomical Measurements

**Figure 1:**
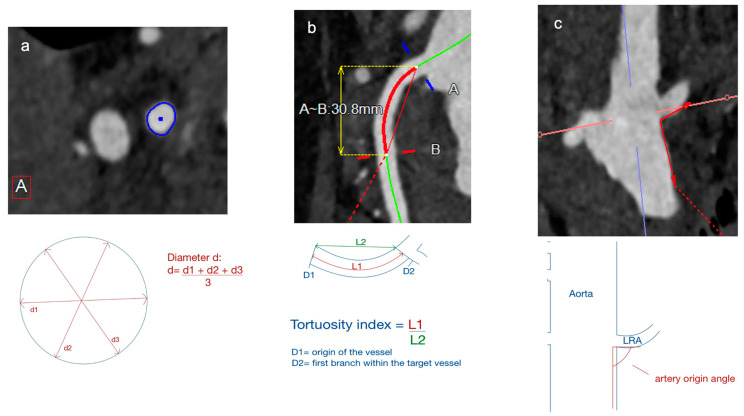
Preoperative target vessel parameter: (**a**) diameter; (**b**) tortuosity; (**c**) artery origin angle. A = Beginning of measurement; B = End of measurement; d1/d2/d3 = Diameter; L1 = Length 1; L2 = Length 2; LRA = Left renal artery.

Pre- and postoperative measurements and case-planning were performed with the Aquarius iNtuition software (Version 4.4, TeraRecon, Foster City, CA, USA). Preoperative measurements of target vessel anatomical characteristics included:

- Diameter: centerline-adjusted automatic measurement (Figure 1).
- Angle: measured as the angle between the target vessel and the aortic wall (Figure 1).
- Tortuosity index: calculated as a ratio between the length along the centerline and the linear distance between the orifice of the target vessel to 3 cm distal into the vessel (Figure 1).

Only fenestrations successfully aligned with the covered stent were included in the post-stenting analysis.

**Figure 2:**
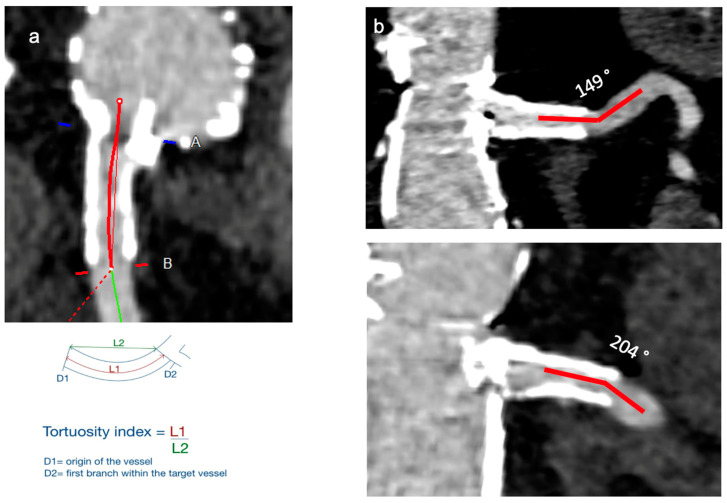
Postoperative target vessel parameter (Part 1): (**a**) tortuosity; (**b**) post-stenting angle. A = Beginning of measurement; B = End of measurement; L1 = Length 1; L2 = Length 2.

**Figure 3:**
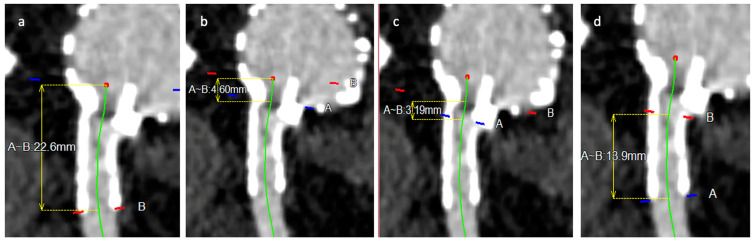
Postoperative target vessel parameter (Part 2): (**a**) total bridging stent length; (**b**) aortic protrusion length; (**c**) bridging length (gap); (**d**) sealing length. A = Beginning of measurement; B = End of measurement.

The target vessel anatomy was assessed on the first postoperative CTA and included the assessment of:

- Change of clock position/horizontal misalignment: difference between planned clock position of branch postoperative changed clock position.
- Tortuosity index: calculated as a ratio between length of bridging stent along the centerline and the linear distance between beginning and end of bridging stent (Figure 2).
- Total bridging stent length: length of stent between the beginning of the branch until the end of stent in target vessel (Figure 3).
- Bridging stent sealing length: length of the stent apposition to the arterial wall into the target vessel (Figure 3).
- Bridging length (gap): length between the end of main body branch and the origin of the target vessel (Figure 3).
- Protrusion: length of bridging stent protrusion from fenestration into aortic lumen (Figure 3).
- Flaring ratio: ratio between diameter of bridging stent in fenestration and diameter of maximum flared stent-graft end in aorta.
- Oversizing ratio: measured as a ratio between the size of native and stented target vessel.
- Post-stenting angle: downward angle between distal stented and proximal native target vessel.

Measurements were independently performed by two staff members of the vascular research team (JS, DB), both qualified vascular surgeons. Significant variations were settled by independent measurements from the senior author (NT).

### 2.2. Endpoints and Definitions

The primary study endpoint was target vessel instability at 12 months. The target vessel instability was defined as occlusion, stenosis and endoleak Type Ic/IIIc and re-intervention. 

Secondary endpoints were target vessels’ instability and major adverse events (MAEs) at 30 days as well as during follow up. 

To assess the impact of preoperative target vessel anatomy (diameter, angle and tortuosity) and postoperative geometry after stenting (change of clock position/horizontal misalignment, total bridging stent length including sealing length, bridging length and protrusion, post-stenting angle, flaring and oversizing ratio) on the target vessel instability, the latter mentioned parameters were analyzed. 

The early postoperative period was defined as occurring within the first 30 days or during hospital stay. Major adverse events included death, acute kidney injury, new-onset dialysis, myocardial infarction, paraplegia, stroke and bowel-ischemia requiring surgical resection and any re-intervention [1]. Technical success was defined as successful delivery of bridging stents to target vessels, and patent target vessels without stenosis or endoleak type Ic/IIIc in first postoperative CTA scan [16]. Computed tomography was performed before discharge or during the first 30 days after the procedure. 

### 2.3. Statistical Analysis

Data analysis was performed with SPSS Statistics (version 28; IBM, Chicago, IL, USA) and R Core Team (2022) (R Foundation for Statistical Computing, Vienna, Austria, https://www.R-project.org/, accessed on 1 March 2024). Continuous variables are expressed as mean ± standard deviation or median with interquartile ranges, according to the normality of distribution. Categorical variables are presented as numbers and percentages. To achieve a more representative analysis, CT and SMA and both renal arteries were divided into two groups (visceral target vessels = VTV; renal target vessels = RTV). 

Cross-tables and *t*-test for independent variables were performed to compare both groups with each other, and for analysis of the impact of anatomical factors and postoperative regimen of antiplatelet or anticoagulation and geometry after stenting on target vessel instability in each group. Covariates with a *p*-value < 0.2 in univariate analysis were entered into the multivariate model. Uni- and multivariate Cox proportional hazards models were used to identify clinical, procedural and anatomical factors associated with target vessel instability. Anatomical parameters that were found to have a significant influence on target vessel instability were further evaluated with the Receiver Operating Characteristics analysis (ROC curve) to determine a cut-off value associated with an increased risk of instability.

To evaluate survival and freedom from re-intervention, analysis was conducted using the Kaplan–Meier survival analysis.

Cumulative incidence rate of target vessel instability with competing risk of death was estimated via the proportional sub-distribution hazards model.

For adequate reporting of freedom from target vessel instability, a combined event of death and target vessel instability was defined. Statistical significance was assessed using the log-rank test (*p* < 0.05).

## 3. Results

A total of 136 consecutive patients including 481 stented target vessels (221 visceral target vessels; 260 renal target vessels) underwent f-EVAR between September 2018 and December 2023 for the treatment of TAAAs and cAAAs. The mean age was 70 ± 10.5 years and 81.6% (111/136) were male. The median aneurysm diameter was 56 mm (IQR 51–65). The indication for treatment included degenerative aneurysms (108/136, 79.4%) and post-dissection aneurysms (28/136, 20.6%). The majority of patients (109/136, 80.1%) were treated electively, and the remainder were performed on in an urgent setting (27/136, 19.9%). Patients’ demographics and characteristics are listed in Table 1.

Of the 136 patients, 106 (77.9%) underwent repair with custom-made fenestrated Cook stent-grafts (Cook medical, Bloomington, NJ, USA) and the remaining 30 patients were treated with physician-modified stent-grafts using the Cook platform (Thoracic Alpha or TX2). Endograft configuration was 1 fenestration and 1 scallop in one case (1%), 2 fenestrations and 1 scallop in 2 cases (1.5%), 3 fenestrations and 1 scallop in 15 cases (11%), 2 fenestrations in 6 cases (4%), 3 fenestrations in 17 cases (12.5%), 4 fenestrations in 88 cases (65%), 5 fenestrations in 6 cases (4%) and 6 fenestrations in 1 case (1%). The main bridging stent-graft used was BeGraft (Bentley InnoMed GmbH, Hechingen, Germany) in 91% of cases and Advanta V12 (Getinge GmbH, Rastatt, Germany) and iCover (iVascular, Freiburg, Germany) were used in the remaining cases. 

In the analysis of pre-, intra- and postoperative parameters between VTV and RTV groups, VTV showed a significantly greater diameter compared to RTV (*p* < 0.001), whereas the artery origin angle and tortuosity index of RTV was greater than in VTV (*p* < 0.001, *p* < 0.001). The diameter and length of used bridging stents were consequently greater in the VTV group (*p* < 0.001, *p* < 0.001). A comparison of both groups is shown in Table S1. 

The technical success rate was 99.2% for all target vessels (478/481). In the VTV group, the technical success rate was 99.1% (219/221) compared to 99.2% (259/260) in the RTV group. Due to celiac trunk stenosis, cannulation was not possible in two patients, but final angiography and postoperative CT scan did not show an endoleak. Due to strut from previous EVAR and stenotic origin, cannulation of a right lower accessory renal artery was not possible. Plug and coiling was performed to prevent endoleak. Perioperative data are shown in Table S2.

### 3.1. 30-Day Outcomes

Within 30 days, seven patients died (5.1%, 7/136), with one death occurring perioperatively after successful endovascular repair due to hemorrhagic shock and consecutive cardiac failure. One patient died during the first postoperative day secondary to extended operating time, resulting in prolonged lower ischemia time and reperfusion syndrome. Another patient died due to multi-organ failure after a complicated course with access bleeding and hemorrhagic shock. One patient died due to myocardial infarction resulting in acute heart failure and lung edema. Respiratory failure was the cause of death in another patient. Due to multi-organ failure, two additional patients died after a complicated postoperative course.

One target vessel instability occurred (0.2%, 1/481) within 30 days. An acute occlusion of a 5 mm BeGraft in a 4 mm right renal artery (RRA) was detected on the postoperative CTA scan due to deterioration of renal function. Successful revascularization with aspiration thrombectomy and relining was performed and kidney function recovered. 

The overall major adverse event rate was 16.2% (22/136). Despite the high rate of acute kidney injury, only two patients needed permanent dialysis. Spinal cord ischemia appeared in six patients. One patient with delayed full paraplegia died during their hospital stay. In the four patients with partial paraplegia, symptoms improved before discharge with supported mobility. In the one patient with immediate complete paraplegia, symptoms remained until discharge.

In fifteen patients (11.0%, 15/136) re-intervention was necessary within 30 days. The majority was due to access-related complications. The complete 30-day outcomes are presented in Table S2. 

### 3.2. Follow-Up Outcomes

The median follow up was 21 months (7–33). In the follow ups, a total of ten target vessel instabilities occurred (2.08%, 10/481). In visceral target vessels, three type IIIc endoleaks occurred in celiac trunk. In renal target vessels, seven instabilities occurred. In the right renal artery three and in left renal artery four instabilities were found (RRA: 2 occlusion, 1 type IIIc endoleak/LRA: 2 occlusions, 1 stenosis, 1 type IIIc endoleak). The superior mesenteric artery (SMA) was not affected. In three cases with renal artery occlusion, the initially deteriorated renal function recovered and no dialysis was necessary. In one case of renal artery occlusion, the patient needed permanent dialysis. Details of target vessel instabilities are shown in Table S3.

**Figure 4:**
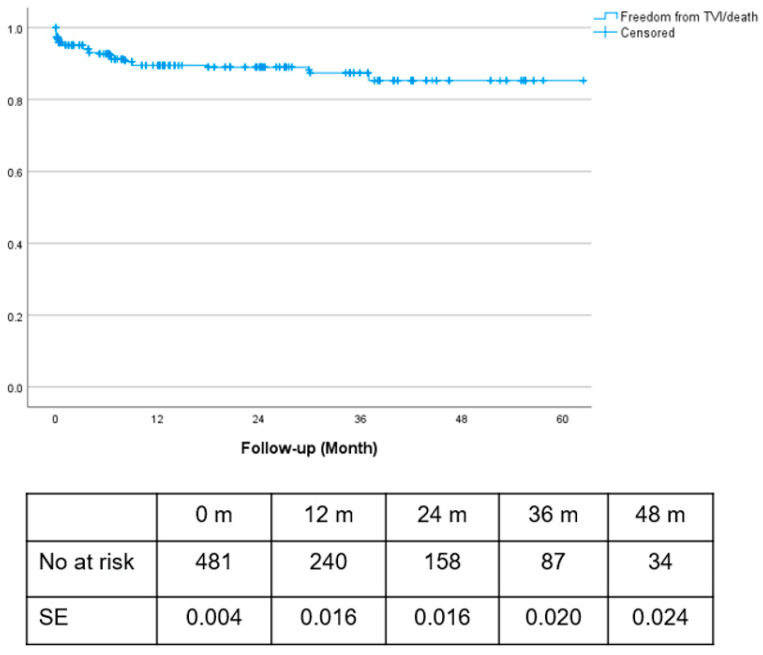
Freedom from composite event (FFCE; death/branch vessel instability).

Estimated freedom from combined events (TV instability/death) was 89.5%, 89% and 85% at 1, 2 and 3 years, respectively (Figure 4).

**Figure 5:**
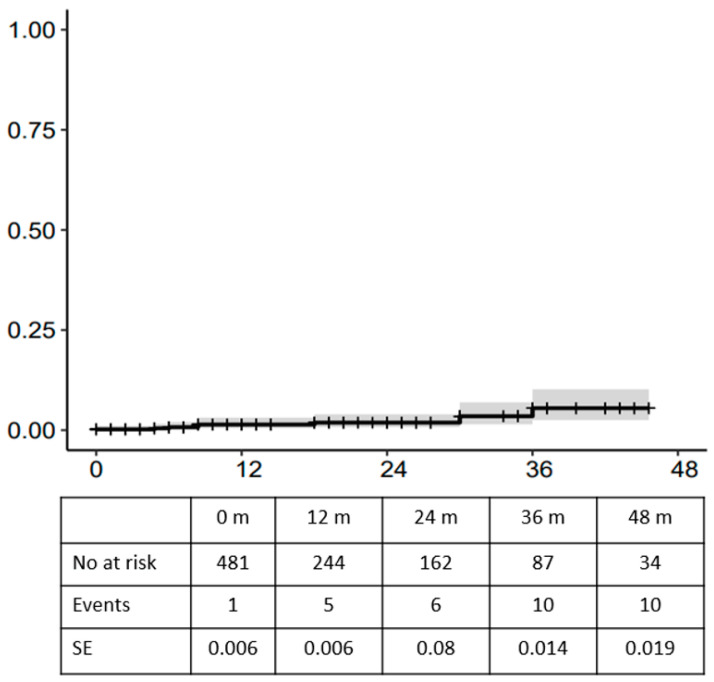
Cumulative incidence (CI) of target vessel instability with death as competing risk.

Cumulative incidence of target vessel instability was 1.4%, 1.8% and 3.4% at 1, 2 and 3 years, respectively (Figure 5).

A total of six patients died after 30 days. Five patients died due to non-aneurysm-related causes and in one case, the cause of death remained unknown. One patient died due to cardiac failure with decompensation resulting in multi-organ failure one month after the procedure. One death was due to recurrent naso-pharyngeal cancer detected four months postoperatively and one death was secondary to metastatic pancreas cancer two years after procedure. One patient suffered from recurrent gastro-intestinal bleeding resulting in hypoxemia and pulseless electric activity and cardiac failure at 9 months after f-EVAR, and two deaths were secondary to COVID, at one and eleven months after the index procedure. Estimated survival was 90% at 1, 2 and 3 years, respectively (Figure S1).

Seventeen patients required re-intervention in follow up (12.5%, 17/136). Follow-up data are summarized in Table S4. The estimated freedom from overall re-intervention was 79%, 73% and 66% at 1, 2 and 3 years, respectively (Figure S2).

### 3.3. Analysis of Target Vessel Instability

An analysis of the influence of patients’ demographics and characteristics, including age, sex, previous aortic intervention, TAAA versus cAAA, maximum aneurysm diameter, post-dissection aneurysm and urgency and postoperative antiplatelet and anticoagulation regimen on target vessel instability was performed. Concerning the univariate analysis, the female sex (HR 1.780, 95% CI 1.048–2.665, *p* = 0.010) showed a significant association with increased target vessel instability. This was also confirmed via a multi-variate analysis (HR 1.217, CI 95% 1.055–1.858, *p* = 0.029). Results of the entire analysis are presented in Table 2. 

Regarding pre-, intra- and postoperative parameters, an analysis of VTV and RTV was performed. In the univariate analysis of the VTV group, a preoperative tortuosity index ≥ 1.25 (HR 15.19, CI 95% 2.50–17.47, *p* = 0.045) and an oversizing ratio ≥ 1.25 (HR 7.739, CI 95% 4.756–12.878, *p* = 0.049) were significantly associated with an increased target vessel instability in uni- and multi-variate analyses (Table 3 and Table S5).

In the RTV group, a target vessel diameter ≤ 4 mm was found to be significantly associated with increased target vessel instability (HR 1.30, CI 95% 1.15–3.26, *p* < 0.001), which was also confirmed in the multi-variate analysis (HR 1.21, CI 95% 1.035–1.274, *p* = 0.009). The diameter of the stent was significantly associated with target vessel insufficiency in uni- but not in multi-variate analysis (HR 1.082, CI% 1.023–3.01, *p* < 0.001). An aortic protrusion of ≥5.75 mm and a stent length of ≥38 mm showed a significant association with target vessel complications in uni- (protrusion: HR 7.06, CI 95% 2.480–10.84, *p* = 0.013; Stent length: HR 1.062, CI 95% 1.031–1.139, *p* = 0.009) and multi-variate analyses (Protrusion: HR 8.21, CI 95% 3.150–12.23, *p* = 0.027; Stent length: HR 1.098, CI 95% 1.027–1.175, *p* = 0.036) (Table 4 and Table S6).

## 4. Discussion

The durability of f-EVAR in complex aortic pathologies is mainly based on patent and sealed target vessels. Secondary interventions after f-EVAR are still frequent and the majority derives from target vessel complications including occlusions, stenosis and endoleaks [5,18,19,20]. Planning of endograft and bridging stents should include the aim of perfect alignment of fenestrations to target vessels and of achieving adequate sealing of bridging stent in fenestration and the target vessel. The size and length of the bridging stent should be adequately chosen to fit the diameter and sealing zone of the target vessel. 

The current study aimed to investigate the influence of pre- and postoperative target vessel anatomy on outcome of f-EVAR in treatment of TAAA and cAAA. As described before, female sex is associated with a higher risk for target vessel instability. This might be explained with the smaller diameter of the vessel diameters seen in female patients. But results have to be interpreted carefully, because only 29% of patients were female.

In the present cohort of patients, a relevant number of TAAA and post-dissection aneurysms have been included. Despite the fact that former studies reported a significant influence of both on target vessel instability, we did not observe these findings in the current study [14].

In the current cohort of patients, the majority of target vessel instabilities were found in renal arteries. The influence of renal arteries in f-EVAR, but more frequently in b-EVAR, is a previously reported issue [21]. A diameter of target vessel ≤ 4 mm was reported as a risk factor for target vessel complications [13,21]. This was also confirmed by the results of the analyzed patients. In clinical practice, treatment of small renal arteries should be evaluated individually. A 4–5 mm renal artery, as a single nutrition vessel of the kidney, should be connected to the main body. But connecting an accessory renal artery with a diameter of 4 mm or below should be weighed against the risk of intraoperative complications and late occlusions. To deal with the challenges of small renal arteries, the standard of practice is to connect target vessels between 4 and 5 mm with a 5–6 mm bridging stent-graft and smoothly attach the stent-graft to the vessel wall with a 6/20 balloon to avoid dissection of the target vessel. 

Squizzato et al. reported an association of a bridging length (gap) ≥ 5 mm with increased target vessel instability [14]. The bridging length might be associated with an increased movement of bridging stent-graft between fenestration and target vessel, leading to damage in the bridging stent-graft ending in endoleak or occlusion. In the observed cohort of patients, bridging length did not show significant differences between patients with and without instabilities. 

As two important factors of bridging stent sealing in the main stent-graft, adequate protrusion and flaring need to be addressed. In the study of Oderich et al., a protrusion length of 3 to 5 mm seemed to guarantee the best results [22]. This was supported by findings of Squizatto et al., who reported a protrusion length below 3 mm as significantly more associated with target vessel instability. It was postulated that a higher risk of disconnection due to short protruding length of bridging stent-graft could be the consequence [14]. As a possible explanation, the extended protrusion might lead to turbulences of blood flow resulting in thrombotic target vessel occlusion. In the current cohort, a protrusion of ≥5.75 mm into the aortic lumen was associated with a significantly higher target vessel instability in the visceral target vessel group. We did not observe any disconnection or occlusion. We only observed one stent-fracture and two insufficencies of sealing in fenestration. 

As an important factor for sealing of bridging stent in fenestration, the role of flaring at the proximal part of the bridging stent was assessed. In the current cohort, the flaring ratio showed no association with target vessel instability. An explanation might be the standardized flaring of the proximal part of the stent-graft with a compliant with a 2 mm bigger balloon than the diameter of the implanted stent. 

To achieve an adequate sealing in the target vessel, sealing length and oversizing of bridging stent are notable parameters. We aim at a sealing length between 10 and 20 mm and an equally or 1 mm greater diameter of the used bridging stent compared to target vessel diameter. In the analyzed patient cohort, sealing length was adequately achieved and showed no difference between patients with or without target vessel instability. In the visceral target vessel group, an oversizing ratio ≥ 1.25 was associated with an increased target vessel instability. This might be due to an increased stress on the intima of the target vessel leading to hyperplasia or local dissection with consecutive stenosis or occlusion. In our cohort, we observed one perioperative dissection of the celiac trunk, which required relining and distal stent extension. But postoperatively, only type IIIc endoleaks occurred in the celiac trunk and no occlusion or stenosis.

A significant correlation with increased preoperative tortuosity and target vessel instability was found in visceral target vessels. The increased tortuosity might lead to a kinking of stent-graft, leading to fracture or/and occlusion. To further examine the influence of kinking of the target vessel after stenting, the post-stenting angle of the transition zone between the end of the stented target vessel and the beginning of the native target vessel was assessed. In the current cohort, no significant association was found.

The findings of the current study contribute to existing knowledge of the geometric and anatomical characteristics which can influence the outcomes of f-EVAR. These characteristics should be taken into account, when planning and deploying endografts during f-EVAR. One of the key challenges is to achieve an adequate bridging distance between fenestration and the target vessel [14]. This issue should be already addressed during sizing and planning. Maybe a selection of a combination of fenestrations and branches could be useful in large aneurysmal sacs, for example, in cases of post-dissection thoraco-abdominal aortic aneurysms (TAAA) [23]. Alternatively, the use of inner branches can be considered in cases with a narrow aorta [24]. Nevertheless, further data are required to validate the optimal approach for cases of f-EVAR with a considerable distance between the endograft and the target vessel.

With the advent of 3D printing technology and the ability to simulate in vitro blood flow, we now have the capability to model pulsatile blood flow under controlled conditions [25]. This advancement enables us to minimize the variability introduced by differing anatomies, which can influence the outcomes. Consequently, these techniques provide a novel approach to accurately predict the behavior of bridging stent-grafts in highly tortuous anatomies and assess potential target vessel instability.

The current study has some notable limitations. It was a single-center retrospective study with a limited number of patients. The low number of events may have limited the power of the statistical analysis, and a longer follow up is necessary for more robust conclusions. To expand the number of patients and events, we also included patients who had less follow-up than initially required for the primary endpoint.

A subgroup analysis concerning anatomical parameters for the TAAA and post-dissection aneurysm was not performed due to the non-significant differences in target vessel instabilities and the small number of events in general, but this should be mentioned as a clear limitation. 

Another limitation is the missing subgroup analysis if there is a difference in vessel anatomy before and after stenting between women and men. We did not perform this due to the limited number of female patients (<20%).

Larger confirmatory multi-center studies may be useful to correlate the specific type of target vessel instability with the postoperative geometric parameters. The study was strengthened by the detailed analysis of target vessel anatomy focusing on the most affected renal arteries. Furthermore, the use of Cox regression analysis allowed us to produce a reliable OR in cases of low numbers of events. 

## 5. Conclusions

Short- and midterm target vessel instability rate in f-EVAR for CAAA and TAAA showed satisfying results in the current cohort of patients. Female sex was associated with an increased risk of target vessel instability. Analysis of pre- and post-stenting target vessel anatomy in f-EVAR showed different findings in target vessel instability between the renal and visceral target vessels. A target vessel diameter ≤ 4 mm and an aortic stent protrusion ≥ 5.75 mm shows a higher risk of target vessel instability in renal target vessels. On the other hand, a preoperative tortuosity index and an oversizing ≥ 1.25 increased the risk of target vessel instability in visceral target vessels.

## Figures and Tables

**Table 1:** Patients’ demographics and characteristics.

	f-EVAR (*n* = 136)
Male	111 (81.6)
Age—years	70 ± 10.5
BMI—kg/cm^2^	26.4 ± 4.6
Hypertension	120 (88.2)
Dyslipidemia	84 (61.8)
CKD	54 (45)
CAD	35 (25.7)
CHF	18 (13.2)
Past PTCA	19 (14)
Past CABG	16 (11.8)
Arrythmia	26 (19.1)
Smoker (current or ex)	97 (71)
COPD	34 (25)
Diabetes	23 (16.9)
PAD	14 (10.3)
Past Stroke/TIA	12 (8.8)
Cancer	15 (10.9)
CTD	3 (2.2)
Previous aortic surgery	
- Open AAA repair - EVAR - TEVAR - FET - Ascending Aortic repair	75 (55.1) 4 (2.9) 12 (8.8) 28 (20.4) 12 (8.8) 19 (13.9)
Degenerative Aneurysm	108 (79)
- Short-Neck	10 (9.3)
- Juxtarenal	71 (65.7)
- Supra/Pararenal	14 (13.0)
- TAAA Crawford Type I	3 (2.8)
- TAAA Crawford Type II	0 (0)
- TAAA Crawford Type III	3 (2.8)
- TAAA Crawford Type IV	5 (4.6)
- TAAA Crawford Type V	2 (1.9)
Post-dissection aneurysm	28 (21)
- Post-Type A Dissection: - TAAA Crawford Type I - TAAA Crawford Type II - TAAA Crawford Type III	16 (57.1) 2 4 5
- Post-Type B Dissection: - TAAA Crawford Type I - TAAA Crawford Type II - TAAA Crawford Type III	12 (42.9) 3 5 4
Urgency	
- Elective	109 (80.1)
- Symptomatic	13 (9.6)
- Rupture	14 (10.3)
Previous aortic surgery	
- Open AAA repair - EVAR - TEVAR - FET - Ascending - Aortic repair	75 (55.1) 4 (2.9) 12 (8.8) 28 (20.4) 12 (8.8) 19 (13.9)

Abbreviations: AAA = Abdominal aortic aneurysm; BMI = Body mass index; CKD = Chronic kidney disease; CAD = Coronary artery disease; CHF = Chronic heart failure; PTCA = Percutaneous transluminal catheter; CABG = Coronary artery bypass; COPD = Chronic obstructive pulmonary disease; PAD = Peripheral artery disease; TIA = Transitory ischemic attack; CTD = tissue disorder; EVAR = Endovascular aortic repair; TEVAR = Thoracic endovascular aortic repair; FET = Frozen elephant technique; TAAA = Thoraco-abdominal aortic aneurysm.

**Table 2:** Uni- and multivariate analysis of patients’ demographic and characteristics (statistics: Cox regression).

	Target Vessel Instability							
N = 136	Yes (*n* = 10)	No (*n* = 126)	Univariate Analysis			Multi-Variate Analysis		
			HR	(CI 95%)	*p*	HR	(CI 95%)	*p*
Sex			1.780	(1.048–2.665)	0.01	1.217	(1.055–1.858)	0.029
Female	5	20						
Male	5	106						
Age	66.3 ± 11.4	70.6 ± 10.4	0.979	(0.923–1.038)	0.480			
Previous surgery			0.408	(0.092–1.802)	0.237			
- No	7	79						
- Yes	3	47						
Aneurysm diameter	51.4 ± 8.9	59.5 ± 13.4	0.948	(0.885–1.014)	0.122	0.966	(0.899–1.038)	0.342
TAAA vs. CAAA	2 8	39 87	1.821	(0.311–10.665)	0.506			
Post-dissection aneurysm			1.63	(0.417–6.38)	0.482			
- No	8	100						
- Yes	2	26						
Urgency			0.562	(0.029–10.951)	0.592			
- Elective	8	98						
- Urgent	2	28						

Abbreviations: cAAA = complex abdominal aortic aneurysm; TAAA = thoraco-abdominal aortic aneurysm.

**Table 3:** Uni- and multi-variate analyses of visceral target vessel instability (statistics: Cox regression).

	Cox Regression					
VTV (N = 221)	Univariate Analysis			Multi-Variate Analysis		
	HR	(CI 95%)	*p*	HR	(CI 95%)	*p*
Preoperative Variables						
Diameter—mm	0.737	(0.331–1.638)	0.454			
Tortuosity index (≥1.25 *)	12.27	(3.011–15.57)	0.039	15.19	(2.50–17.47)	0.045
Artery origin angle—°	1.023	(0.984–1.064)	0.252			
Intraoperative Variables						
Number of stents	0.048	(0.027–2.25)	0.920			
Diameter stent	1.185	(0.384–3.654)	0.768			
Postoperative variables						
Stent length—mm	0.749	(0.506–1.109)	0.148			
Tortuosity index	4.399	(1.46–6.07)	0.806			
Sealing length—mm	0.809	(0.538–1.218)	0.310			
Bridging length—mm	0.754	(0.034–16.797)	0.858			
Aortic protrusion—mm	1.355	(0.349–5.261)	0.661			
Flaring ratio	0.025	(0.010–20.750)	0.282			
Post-stenting angle—°	0.981	(0.949–1.014)	0.263			
Oversizing Ratio (≥1.25 *)	11.49	(5.865–22.545)	0.009	7.739	(4.756–12.878)	0.049

* Cut-off values are calculated using the receiver operating characteristics analysis.

**Table 4:** Uni- and multi-variate analyses of renal target vessel instability (statistics: Cox regression).

RTV (N = 260)	Univariate Analysis			Multi-Variate Analysis		
	HR	(CI 95%)	*p*	HR	(CI 95%)	*p*
Preoperative Variables						
Diameter—mm (<4 mm *)	1.30	(1.15–3.26)	<0.001	1.21	(1.035–1.274)	0.009
Tortuosity index	4.419	(0.003–7.336)	0.694			
Angle—°	1.013	(0.986–1.041)	0.337			
Intraoperative Variables						
Number of stents	0.138	(0.013–5.37)	0.639			
Diameter stent	1.082	(1.023–3.01)	<0.001			
Postoperative variables						
Stent length—mm (≥38 mm *)	1.062	(1.031–1.139)	0.009	1.098	(1.027–1.175)	0.036
Tortuosity index	2.55	(0.05–12.24)	0.453			
Sealing length—mm	1.014	(0.918–1.121)	0.778			
Bridging length—mm	1.101	(0.897–1.351)	0.356			
Aortic protrusion—mm (≥5.75 *)	7.06	(2.480–10.84)	0.013	8.21	(3.150–12.23)	0.027
Flaring ratio	0.107	(0.002–6.372)	0.284			
Post-stenting angle—°	1.019	(0.992–1.047)	0.170			
Oversizing Ratio	3.238	(0.179–58.456)	0.426			

* Cut-off values are calculated using the receiver operating characteristics analysis.

## Data Availability

Raw data were generated at Ludwig-Maximilian-University Munich Department of Vascular Surgery. Derived data supporting the findings of this study are available from Daniel Becker on request.

## Supplementary Materials

The following supporting information can be downloaded at: https://www.mdpi.com/article/10.3390/jcm13102898/s1, Figure S1: Survival after fenestrated endovascular aortic repair; Figure S2: Freedom from re-intervention; Table S1: Perioperative and 30-days data; Table S2: Follow up data; Table S3: Details of target vessel instabilities; Table S4: Comparison of target vessel anatomy and post-stenting geometry between renal and visceral target vessels (Statistics: *t*-test for independent samples); Table S5: Univariate analysis of visceral target vessel instability (Statistics: *t*-test for independent variables); Table S6: Univariate analysis of renal target vessel instability (Statistics: *t*-test for independent variables).
