# Supplementary material for: Analysis of Target Vessel Instability in Fenestrated Endovascular Repair (f-EVAR) in Thoraco-Abdominal Aortic Pathologies
Source: J Clin Med. 2024 May 14;13(10):2898. doi: 10.3390/jcm13102898 (PMC11122549; doi:10.3390/jcm13102898)
Supplement: Supplementary file 1 [file jcm-13-02898-s001.zip › jcm-2965096-supplementary.pdf]

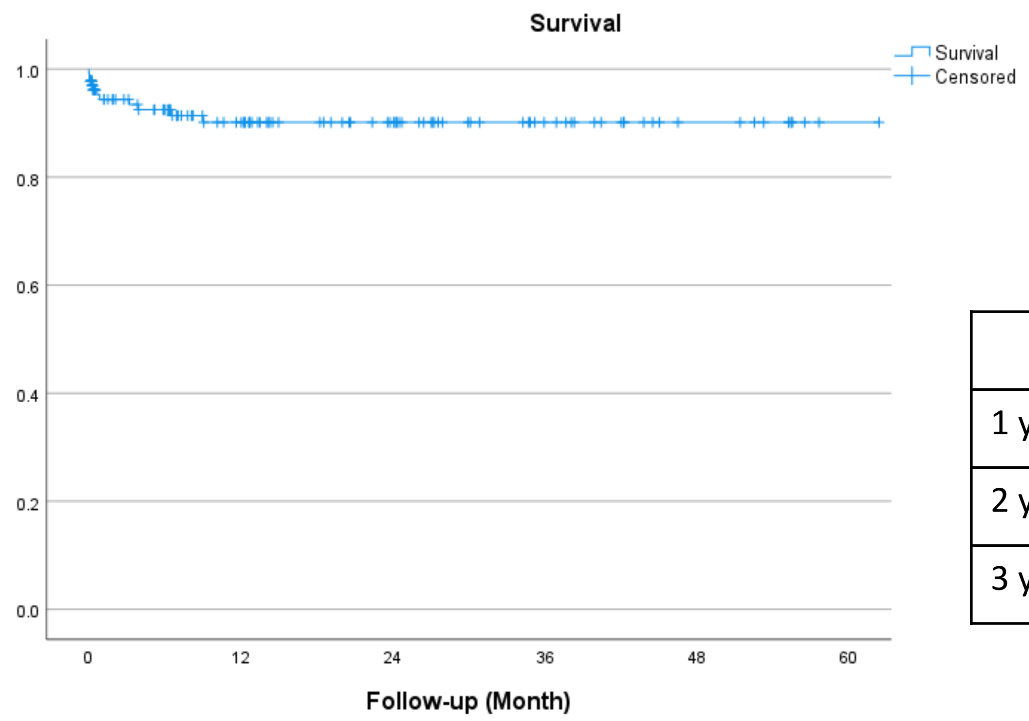

	Survival
1 yr	90 %
2 yrs	90 %
3 yrs	90 %

	0 m	12 m	24 m	36 m	48 m
No at risk	136	68	44	23	10
SE	0.007	0.029	0.029	0.029	0.029

Figure S1: Survival after fenestrated endovascular aortic repair

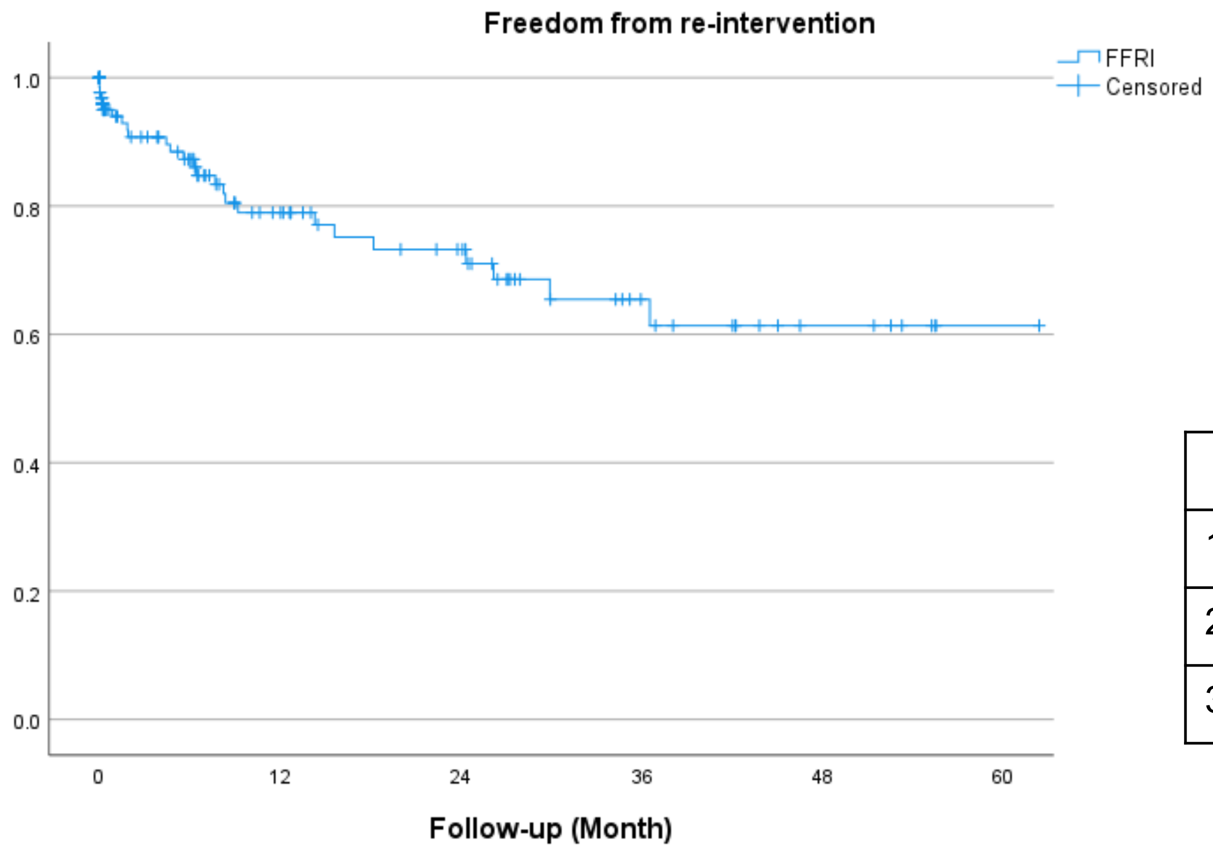

	FFRI
1 yr	79 %
2 yrs	73 %
3 yrs	66 %

	0 m	12 m	24 m	36 m	48 m
No at risk	136	49	35	16	7
SE	0.010	0.044	0.052	0.063	0.071

Figure S2: Freedom from re-intervention

Table S1: Perioperative and 30-days data

	n=136
	n (%)
General anesthesia	136 (100)
CSFD	44 (32.4)
Upper extremity approach	16 (11.8)
- Rigth side	
- Left side	14 (87.5)
	2 (22.5)
Percutaneous femoral	136 (100)
Staged repair	52 (38.2)
Contrast volume - ml	214 (170 - 300)
Fluoroscopy time - min	69 (51 - 93)
Radiation dose – $cGycm^2$	2607 (1609 – 4427)
Operating time - min	228 (194 – 282)
Technical success	500/504 (92.3)
Length of hospital stay - days	8 (7 - 12)
Length of ICU stay - days	2 (1 – 3)
Any MAE	22 (16.2)
Acute kidney injury	13 (36.1)
- New onset of dialysis:	3 (23.1)
- Temporary	1 (7.7)
- Permanent	2 (15.4)
SCI	6 (16.7)
- Immediate partial	2 (33.3)
- Immediate full	1 (17.7)
- Delayed partial	2 (33.3)
- Delayed full	1 (17.7)
Bowel ischemia w/resection	1 (2.8)
Minor stroke	1 (2.8)
Myocardial infarction	1 (2.8)
30-day mortality	7 (5.1)
30-day re-intervention	15 (11.0)
- Retrograde Typ A Diss	1 (6.7)
- Target vessel occlusion	1 (6.7)
- Limb ischemia	1 (6.7)
- Access false aneurysm / bleeding	
- Lymph fistula	7 (46.6)
- Other	2 (13.3)
	3 (20.0)

(Abbreviation: ICU=Intensive care unit, IQR=Interquartile range, MAE=Major adverse events, SCI=Spinal cord ischemia)

Supplementary Table S2: Follow up data

	f-EVAR<br><br>(n=136)
	n (%)
Follow up – month, median, IQR	21 (7-33)
Follow-up mortality	13 (9.6)
Follow up re-intervention	17 (12.5)
- TV related (details below):	10 (58.8)
- Graft related:	7 (41.2)
- EL Ia	1 (14.3)
- EL Ib	
- Limb occlusion/stenosis	3 (42.9)
- Prothesis infection	
	2 (28.6)
	1 (14.3)
Follow up TV instability	10 (7.4)
- Occlusion	4 (40.0)
- Stenosis	1 (10.0)
- EL I/III	5 (50.0)

(Abbreviation: EL=Endoleak, TV=Target vessel, SD=Standard deviation)

Supplementary Table S3: Details of target vessel instabilities

Index operation	Indication	Diameter target vessel	Bridging stent	Indication/TVI	Time	Treatment	Graft
4 x CMD	Juxtarenal AAA	LRA: 4 mm	6/22 Be Graft	LRA: Occlusion	18 months	Thrombaspiration/PTA/ Stening	6/22 BeGraft
4 x CMD	Suprarenal AAA	RRA: 3 mm/ LRA: 5 mm LRA	RRA: 5/27 BeGraft, LRA: 6/38 BeGraft	RRA: Occlusion/ LRA: EL IIIc	37 months	Re-Lining LRA/Unsuccesful revasc. RRA	2 x 6/38 BeGraft
4 x CMD	Juxtarenal AAA	LRA: 4 mm	6/22 Advanta	LRA: Stenosis	5 months	PTA	None
4 x CMD	Juxtarenal AAA	LRA: 3 mm	5/27 BeGraft	LRA: Occlusion	8 months	Thrombaspiration/PTA/ Stentin	5/27 Begraft
4 x CMD	Juxtarenal AAA	RRA: 4 mm	6/22 Advanta	RRA: Occlusion	0 month	Thrombaspiration/PTA/ Stenting	6/27 BeGraft
4 x CMD	Type II Post dissection aneurysm	RRA: 6 mm	7/22 BeGraft	RRA: Type III c EL	2 months	Re-Lining	2 x 7/22 BeGraft
4 x CMD	Type II Post Dissection aneurysm	CT: 9 mm	10/27 BeGraft	CT: Type IIIc EL	30 months	Re-Lining	2 x 10/27 BeGraft
4 x CMD	Juxtarenal AAA	CT: 6 mm	8/27 BeGraft	CT: Type IIIc EL	8 months	PTA	None
4 x SMD	Suprarenal AAA	CT: 6 mm	8/27 BeGraft	CT: Type IIIc EL	6 months	PTA	None

(Abbreviation: CT=Celiac trunk, EL=Endoleak, LRA=Left renal artery, PTA=Percutaneous transluminal angioplasty, RRA=Rigth renal artery, TVI=Target vessel instability)

Supplementary Table S4: Comparison of target vessel anatomy and post-stenting geometry between renal and visceral target vessels (Statistics: *t*-test for independent samples)

N=481	Renal target vessels (N=260)	Visceral target vessels (N=221)	p
Diameter – mm	5.8 ± 1.1	7.9 ± 1.5	<0.001
Artery origin angle - °	73.1 ± 23.2	54.5 ± 21.8	<0.001
Tortuosity index - Orifice to 3 cm	1.09 ± 0.09	1.07 ± 0.07	<0.001
Number of stents	1.017 ± 0.18	2.0 ± 0.0	<0.001
Diameter stent – mm	7.3 ± 1.24	8.21 ± 1.06	<0.001
Length stent – mm	27.5 ± 6.17	29.7 ± 73	<0.001
Change of clock position - min	17.27 ± 17.08	15.86 ± 16.28	0.207
Tortuosity index	1.05 ± 0.05	1.05 ± 0.07	0.644
Protrusion - mm	4.05 ± 1.0	4.10 ± 0.99	0.584
Sealing length – mm	17.76 ± 7.74	17.52 ± 7.27	0.741
Bridging length – mm	2.46 ± 2.21	2.34 ± 1.73	0.521
Post-stenting angle - °	180.10 ± 30.52	182.68 ± 32.30	0.394
Flaring ratio	1.32 ± 0.17	1.30 ± 0.16	0.292
Oversizing Ratio	1.13 ± 0.23	1.10 ± 0.17	0.230

(Values are presented using mean and standard deviation after testing of normal distribution)

Supplementary Table S5: Univariate analysis of visceral target vessel instability (Statistics: *t*-test for independent variables)

VTV	Total (n = 221)	Target Vessel Instability		p
		Yes (n = 3)	No (n =218)	
Preoperative variables				
Diameter – mm	7.87 ± 1.49	7.33 ± 1.52	7.88 ± 1.5	0.531
Tortuosity index	1.07 ± 0.07	1.25 ± 0.63	1.07 ± 0.66	<0.001
Angle - °	54.49 ± 21.79	73.33 ± 12.58	54.23 ± 21.79	0.132
Intraoperative variables				
Number of stents	1.03 ± 0.20	1.00 ± 0.00	1.03 ± 0.21	0.777
Stent length – mm	29.72 ± 8.5	25.67 ± 2.30	29.79 ± 8.58	0.408
Diameter stent - mm	8.21 ± 1.06	8.33 ± 1.52	8.20 ± 1.05	0.841
Postoperative variables				
Change of clock position - min	15.86 ± 16.28	8.33 ± 1.52	15.99 ± 16.37	0.421
Tortuosity index	1.05 ± 0.07	1.06 ± 0.06	1.04 ± 0.07	0.715
Sealing length – mm	17.52 ± 7.27	14.33 ± 2.51	17.57 ± 7.31	0.446
Bridging length – mm	2.34 ± 1.73	2.00 ± 0.0	2.34 ± 1.74	0.737
Protrusion	4.10 ± 0.99	4.33 ± 0.58	4.09 ± 0.99	0.686
Flaring ratio	1.30 ± 0.16	1.25 ± 0.51	1.31 ± 0.16	0.597
Post-stenting angle - °	182.68 ± 32.30	160 ± 53.4	183.03 ± 31.97	0.221
Oversizing ratio	1.099 ± 0.16	1.37 ± 0.55	1.09 ± 0.15	0.004
Postoperative medication				
Aspirin	193 (87.7%)	3 (100%)	190 (87.6%)	1.000
Clopidogrel	109 (49.8%)	3 (100%)	106 (49.1%)	0.122
Warfarin	15 (6.8%)	0 (0%)	15 (6.9%)	1.000
DOAC	23 (10.5%)	0 (0%)	23 (10.6%)	1.000
Enoxaparin	8 (3.7%)	0 (0%)	8 (3.7%)	1.000

(Values are presented using mean and standard deviation after testing of normal distribution)

Supplementary Table S6: Univariate analysis of visceral target vessel instability (Statistics: *t*-test for independent variables)

RTV	Total (n = 260)	Target Vessel Instability		p
		Yes (n = 7)	No (n =253)	
Preoperative variables				
Diameter – mm	5.84 ± 1.13	3.86 ± 0.38	5.89 ± 1.09	<0.001
Tortuosity index	1.10 ± 0.09	1.11 ± 0.09	1.10 ± 0.09	0.763
Angle - °	73.10 ± 23.23	83.29 ± 36.5	72.82 ± 22.8	0.241
Intraoperative variables				
Number of stents	1.017 ± 0.18	1.00 ± 0.0	1.02 ± 0.18	0.802
Stent length – mm	27.49 ± 6.16	32 ± 16.11	27.34 ± 5.64	0.023
Diameter stent - mm	7.28 ± 1.24	5.57 ± 0.53	7.33 ± 1.21	<0.001
Postoperative variables				
Change of clock position – min	17.27 ± 17.09	10.71 ± 11.33	17.50 ± 17.22	0.303
Tortuosity index	1.05 ± 0.05	1.07 ± 0.07	1.04 ± 0.05	0.314
Sealing length – mm	17.76 ± 7.74	18.57 ± 7.85	17.73 ± 7.75	0.779
Bridging length – mm	2.46 ± 2.21	2.86 ± 1.21	2.45 ± 2.24	0.631
Protrusion – mm	4.05 ± 1.00	5.86 ± 0.38	3.99 ± 0.96	<0.001
Flaring ratio	1.32 ± 0.17	1.29 ± 0.18	1.32 ± 0.17	0.658
Post-stenting angle - °	180.10 ± 30.52	195.86 ± 20.16	179.61 ± 30.69	0.166
Oversizing ratio	1.13 ± 0.23	1.20 ± 0.36	1.12 ± 0.22	0.369
Postoperative medication				
Aspirin	228 (88.4%)	5 (71.4%)	223 (88.8%)	0.190
Clopidogrel	123 (47.7%)	2 (28.6%)	121 (48.2%)	0.450
Warfarin	18 (7.0%)	2 (28.6%)	16 (6.4%)	0.078
DOAC	26 (10.1%)	0 (0%)	26 (10.4%)	1.000
Enoxaparin	8 (3.1%)	0 (0%)	8 (3.2%)	1.000

(Values are presented using mean and standard deviation after testing of normal distribution)
